# Treatment of Restless Legs Syndrome in Dialysis Patients

**DOI:** 10.1111/sdi.70040

**Published:** 2026-07-15

**Authors:** Kenji Sakurai, Yoshitaka Kurihara, Hiromi Hosoya, Fumi Yamouchi, Takeshi Saito

**Affiliations:** ^1^ Dialysis Center Hashimoto Clinic Sagamihara Kanagawa Japan

**Keywords:** α1‐microglobulin, antioxidant property, restless legs syndrome

1

We wish to provide an academic commentary on the article “Utility of Adding Once‐Weekly Hemoadsorption to High‐Volume Hemodiafiltration for Refractory Restless Legs Syndrome: A Clinical Case Report” by Pedreros‐Roosales et al. [1]. The authors presented a dialysis patient with severe, treatment‐refractory restless legs syndrome (RLS) whose symptoms resolved following the addition of once‐weekly hemoperfusion using the Jafron HA130 cartridge (HA130) to thrice‐weekly high‐volume hemodiafiltration (Hv‐HDF). They emphasized that a blood flow rate of ≥ 400 mL/min is preferable for achieving efficient clearance of β2‐microglobulin (B2M, 11.8 kDa), a representative marker of middle‐molecules removal and one of the prognostic indicators in dialysis populations. On this basis, they proposed that the combined Hv‐HDF‐plus‐HA130 strategy may have broader applicability for other uremic manifestations.

The pathophysiology of primary RLS is multifactorial, involving the accumulation of middle and large molecules, dopaminergic dysfunction, iron deficiency, genetic predisposition, and lifestyle factors. In contrast, RLS in dialysis patients often reflects a distinct and more refractory mechanism. Thus, the rapid decline in the IRLS score from 32 to 0 within 3 weeks represents an exceptional therapeutic response.

In 2013, we reported at the 50th ERA‐EDTA Congress that RLS symptoms resolved when α1‐microglobulin (A1M, 33 kDa) was removed with high efficiency (Table 1) [2]. In the present re‐evaluation of those data, we examined whether reduction rates of B2M or A1M were associated with clinical improvement. Our analysis demonstrated a strong correlation between A1M removal and symptom alleviation, indicating that enhanced A1M clearance is closely linked to RLS resolution (Figure 1). Furthermore, in 2020, we documented two additional cases of recurrent, refractory RLS that improved with sustained high‐efficiency online HDF achieving A1M reduction rates ≥ 40% [3].

**TABLE 1:** Clinical course of restless legs syndrome in seven dialysis patients.

Case 1	Dialysis method	FDY: HD	FDY: PreHDF 40 L	PES‐Dα: HD	
	IRLS R scale score	31	0	21	
	Sp Kt/V	2.1	2.0	2.4	
	B2M RR (%)	74.4	79.5	77.6	
	A1M RR (%)	31.6	42.7	27.5	
Case 2	Dialysis method	FDY: PreHDF 30 L	APS‐E: HD	FDY: PreHDF 30 L	
	IRLS R scale score	0	30	0	
	Sp Kt/V	1.6	1.7	1.7	
	B2M RR (%)	84.7	77.5	82.4	
	A1M RR (%)	48.7	28.8	41.9	
Case 3	Dialysis method	FDY: PreHDF 40 L	FDY: HD	PES‐Dα: HD	FDY: PreHDF 50 L
	IRLS R scale score	0	32	18	0
	Sp Kt/V	1.8	2.8	2.4	1.9
	B2M RR (%)	83.1	82.0	82.9	85.9
	A1M RR (%)	39.1	29.9	33.5	41.9
Case 4	Dialysis method	FDY: HD	FDY: Pre‐HDF 50 L	PES‐Dα: HD	
	IRLS R scale score	37	13	22	
	Sp Kt/V	1.7	1.8	2.1	
	B2M RR (%)	66.1	74.4	65.1	
	A1M RR (%)	26.2	34.6	22.7	
Case 5	Dialysis method	FDY: PostHDF 8 L	ABH: PostHDF 6 L	FDY: PostHDF 6 L	
	IRLS R scale score	14	22	0	
	Sp Kt/V	1.3	1.3	1.5	
	B2M RR (%)	69.8	69.4	72.9	
	A1M RR (%)	30.7	21.6	44.2	
Case 6	Dialysis method	PES‐Dα: HD	FDY: Pre‐HDF 35 L	FDY: PreHDF 50 L	FDY: PreHDF 60 L
	IRLS R scale score	25	19	6	0
	Sp Kt/V	1.4	1.3	1.2	1.2
	B2M RR (%)	72.2	77.6	78.2	78.7
	A1M RR (%)	23.8	36.5	36.1	41.5
Case 7	Dialysis method	FDY: HD	MFX: PreHDF 60 L	MFX: PreHDF 50 L	
	IRLS R scale score	32	18	0	
	Sp Kt/V	1.5	1.5	1.6	
	B2M RR (%)	73.6	81.6	81.7	
	A1M RR (%)	24.0	47.5	43.4	

*Note:* IRLS rating scale score: 0, none; 1–10, mild; 11–20, moderate; 21–30 severe; 31–40, very severe.

**FIGURE 1:**
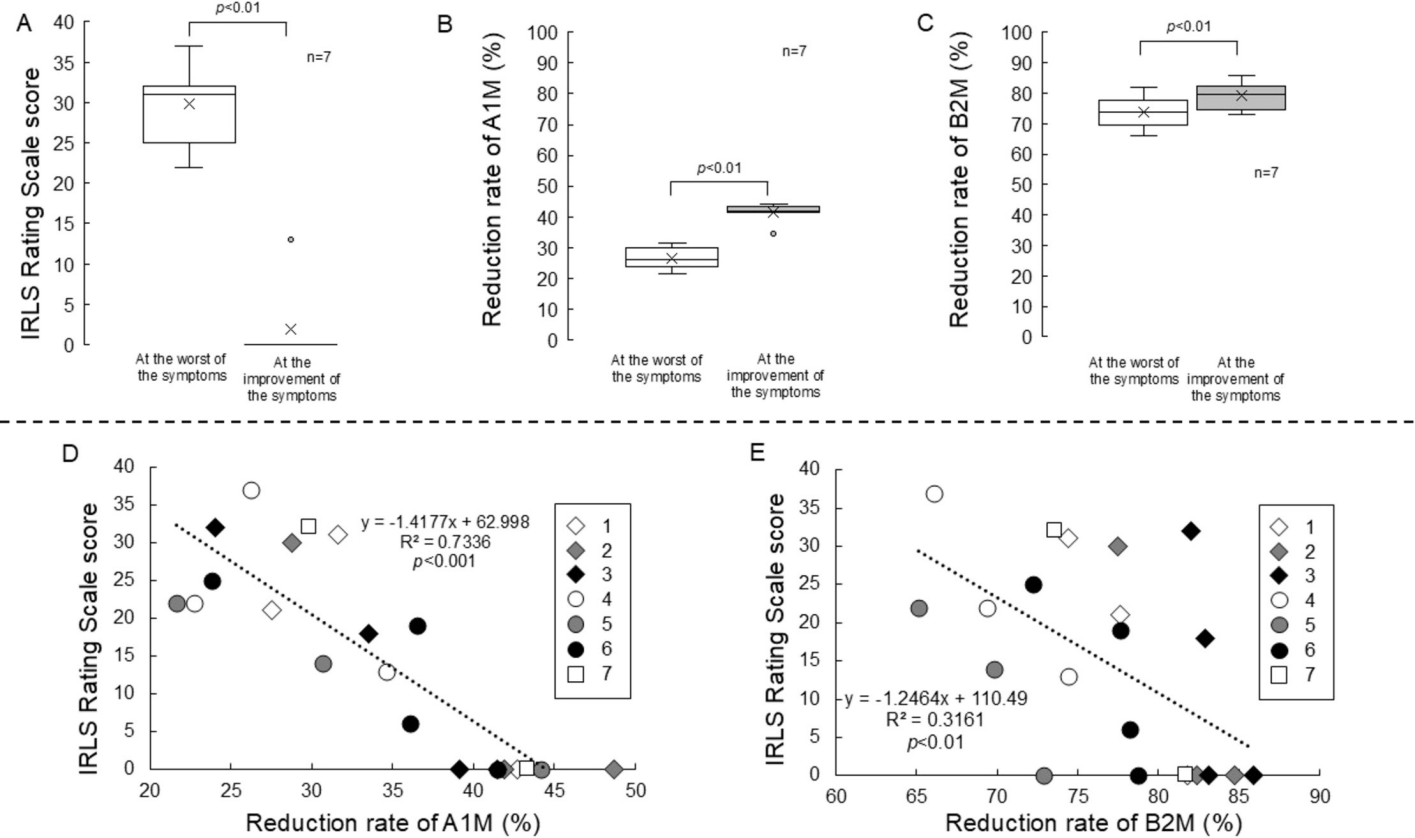
Clinical data from seven patients with restless legs syndrome (RLS), previously reported elsewhere [2], were retrospectively analyzed. First, the IRLS Rating Scale score (IRLS score) (A) and the reduction rates of α1‐microglobulin (A1M) (B) and β2‐microglobulin (B2M) (C) were compared between periods of symptom exacerbation and improvement. During exacerbation, the IRLS score was significantly higher, whereas the reduction rates of A1M and B2M were significantly lower than during improvement (*n* = 7, Wilcoxon signed‐rank test), suggesting reduced removal efficiency during severe RLS symptoms. Second, longitudinal clinical data from the same patients (2–4 data points each) were used to assess correlations between the reduction rates of A1M or B2M and the IRLS score using *r* ^2^ and Spearman's rank correlation coefficient. The reduction rate of A1M (D) showed a stronger correlation with the IRLS score than B2M (E), suggesting that RLS severity is more closely associated with A1M removal efficiency [2].

A1M possesses antioxidant properties, including heme scavenging, inhibition of oxidative reactions, and enzymatic reduction of reactive oxidative intermediates [4, 5]. We hypothesize that, unlike primary RLS, dialysis‐related RLS may arise from diffuse radical‐mediated inflammation of glial cells surrounding synapses of multiple primary afferent fibers (Aβ, Aδ, and C fibers), generating characteristic dysesthesias such as creeping sensations, tingling, and irritability. Because radicals contribute to the inflammation of glial cells, accelerated A1M turnover may mitigate oxidative stress, attenuate neuroinflammation, and consequently ameliorate symptoms. We propose the following mechanistic sequence: efficient removal of degraded A1M by HDF; compensatory hepatic upregulation of newly synthesized A1M; antioxidant and radical‐scavenging activity of this newly produced A1M; and subsequent symptom improvement [4, 5].

On the other hand, HA130 adsorbs protein‐bound toxins, inflammatory cytokines, and middle to large molecules due to the manufacturer's specifications. So we consider that combining Hv‐HDF at high blood flow with HA130 hemoperfusion may reduce persistent inflammation of the glial cells in the dorsal horn, ultimately contributing to RLS resolution.

## Conflicts of Interest

The authors declare no conflicts of interest.

## Data Availability

The data that support the findings of this study are available from the corresponding author upon reasonable request.
